# Psychosocial Distress Patterns and Their Associations With Deliberate Self‐Harm and Suicidality: A Latent Profile Analysis Among Chinese University Students

**DOI:** 10.1155/da/4001851

**Published:** 2026-07-27

**Authors:** Anqi Xiong, Yan Huang, Yi Yang, Yuan Li, Siqi Xiong, Yanxing Yu, Biru Luo, Shujuan Liao

**Affiliations:** ^1^ Department of Nursing, West China Second University Hospital, Sichuan University, Chengdu, Sichuan, China/West China School of Nursing, Sichuan University, Chengdu, Sichuan, China, scu.edu.cn; ^2^ Key Laboratory of Birth Defects and Related Diseases of Women and Children (Sichuan University), Ministry of Education, Chengdu, Sichuan Province, China, moe.edu.cn; ^3^ College of Life Science, Leshan Normal University, Leshan, Sichuan, China, lsnu.edu.cn; ^4^ School of Nursing, Chengdu Medical College, Chengdu, China, cmc.edu.cn; ^5^ Qingdao Medical College, Qingdao University, Qingdao, Shandong, China, qdu.edu.cn

**Keywords:** deliberate self-harm, latent profile analysis, psychosocial distress, suicidality, university students

## Abstract

**Background:**

Deliberate self‐harm (DSH) and suicidality are critical public health concerns among university students, a group facing unique psychosocial stressors. While existing literature has revealed the variable‐centered characteristics of psychosocial distress and DSH within this population, a person‐centered perspective provides a more nuanced understanding of individual differences, which helps inform the development of targeted interventions.

**Objectives:**

This study aims to identify distinct psychosocial distress profiles among Chinese university students, examine their associations with DSH and suicidality, and explore the sociodemographic factors that influence these profiles.

**Methods:**

A nationwide cross‐sectional survey was conducted among 30,992 Chinese university students. Psychosocial distress was assessed using validated scales for loneliness, depressive symptoms, sleep quality, and problematic internet use (PIU). Latent profile analysis (LPA) was employed to identify psychosocial distress profiles. The Bolck–Croon–Hagenaars (BCH) method was applied to compare DSH and suicidality across these profiles, and the R3STEP approach was used to examine sociodemographic correlates of profile membership.

**Results:**

Three distinct psychosocial distress profiles were identified: Class 1 (minimal distress profile, 60.08%), Class 2 (moderate distress profile, 34.72%), and Class 3 (severe distress profile, 5.20%). Participants in Class 3 reported significantly higher levels of DSH (*M* = 7.47) and suicidality (*M* = 1.98) than those in Class 2 (DSH: *M* = 2.10, suicidality: *M* = 0.44) and Class 1 (DSH: *M* = 0.32, suicidality: *M* = 0.05) (all *p* < 0.001). Sociodemographic characteristics, including screen time, exercise duration, enrolled program, romantic relationship status, number of romantic relationships, and sexual experience, differed significantly across profiles (all *p* < 0.001).

**Conclusion:**

Based on the selected latent profile solution, approximately 40% of Chinese university students were classified as experiencing elevated psychosocial distress. These profiles were associated with increased levels of DSH and suicidality. Tailored interventions informed by psychosocial distress profiles and sociodemographic characteristics may help improve health management and risk reduction. Given the cross‐sectional design, temporal relationships cannot be established, and reverse causation cannot be ruled out. Future longitudinal and interventional studies are needed to validate these findings and facilitate their translation into practice.

## 1. Introduction

University students are particularly susceptible to psychosocial distress due to significant developmental transitions, such as adapting to independent living and managing academic demands [1]. This vulnerability may be further compounded by unique stressors such as separation from family, academic‐extracurricular conflicts, and uncertainty regarding future careers [2]. Psychosocial distress in this population often manifests as emotional difficulties, including loneliness and depressive symptoms [3], as well as behavioral issues such as poor sleep quality and problematic internet use (PIU) [4, 5]. These distress factors often co‐occur and may interact, potentially contributing to emotional dysregulation, interpersonal difficulties, and maladaptive coping patterns [6].

Deliberate self‐harm (DSH) and suicidality are critical global public health concerns that frequently co‐occur and are consistently associated with elevated levels of psychosocial distress [7]. These behaviors are among the leading causes of death among individuals aged 15–29 years worldwide [8, 9]. University students may be particularly vulnerable due to the high prevalence of unmet mental health needs and delays in help‐seeking [10]. Identifying psychosocial distress patterns in this population is crucial for early detection and intervention, offering significant societal and clinical benefits while mitigating the long‐term impact of mental health challenges [10].

Although previous studies have examined psychosocial distress in relation to DSH and suicidality [3, 11], most have adopted a reductionist, variable‐centered approach, focusing on isolated variables such as depressive symptoms or loneliness [11, 12]. From a conceptual perspective, variable‐centered approaches examine associations among variables across individuals and generally assume population homogeneity, which may obscure meaningful subgroup differences [13]. In contrast, person‐centered approaches seek to identify latent subpopulations characterized by distinct configurations of multiple indicators, thereby explicitly modeling population heterogeneity [14, 15]. This distinction is particularly relevant in the context of psychosocial distress, which is inherently multidimensional and often involves co‐occurring and interacting symptoms [15]. Accordingly, a person‐centered approach may provide a more nuanced understanding of how different constellations of distress relate to DSH and suicidality [16].

Latent profile analysis (LPA) is a widely used person‐centered method that enables the identification of subgroups based on multiple continuous indicators. Although LPA has been applied to classify individuals based on single domains, such as emotional regulation or DSH behaviors [17, 18], relatively few studies have simultaneously incorporated both emotional and behavioral indicators to characterize psychosocial distress profiles among university students [19]. Moreover, while sociodemographic factors have been extensively examined in relation to psychological distress, their associations with latent psychosocial distress profiles remain insufficiently explored within person‐centered frameworks. This gap may limit the understanding of heterogeneity in distress patterns and constrain the development of targeted intervention strategies.

To address these limitations, our study employed LPA to identify distinct psychosocial distress profiles among Chinese university students and to examine their associations with DSH, suicidality, and sociodemographic characteristics. The conceptual framework was informed by the interpersonal theory of suicide (ITS), which proposes that suicidal desire is linked to thwarted belongingness and perceived burdensomeness, alongside hopelessness regarding change in these states [20]. Within this framework, the selected psychosocial indicators may be conceptually related to key ITS constructs. Specifically, loneliness may be associated with thwarted belongingness through perceived deficits in meaningful social connection [21], whereas depressive symptoms may relate to perceived burdensomeness through feelings of worthlessness and self‐perceived burdens [22]. Poor sleep quality may contribute to interpersonal disconnection by impairing socio‐emotional functioning [23]. PIU may exacerbate interpersonal disconnection and difficulties in psychosocial functioning, potentially reinforcing perceived burdensomeness and hopelessness [24].

Guided by this theoretical framework, we conducted a nationwide cross‐sectional survey to explore how the interplay of loneliness, depressive symptoms, sleep quality, and PIU contributes to DSH and suicidality. Specifically, this study aimed to (1) identify distinct psychosocial distress profiles using LPA; (2) examine associations between these profiles and DSH and suicidality; and (3) explore sociodemographic factors influencing variations in psychosocial distress. The findings of this study may help inform the development of targeted and context‐sensitive mental health strategies for university students.

## 2. Methods

We used the STROBE checklist to improve the rigor of the study design and reporting quality.

### 2.1. Study Design and Setting

This was a nationwide cross‐sectional study conducted from April to July 2024. To ensure broad geographic representation, two public universities were selected from each of the seven major regions in China (Northeast, North, Central, East, South, Northwest, and Southwest), yielding a total of 14 institutions. Within each university, participants were recruited using a convenience sampling approach through institutional online platforms and student networks.

### 2.2. Participants

The study recruited university students from 14 public universities in China. Participants were invited to complete an online questionnaire if they (1) were Chinese nationality; (2) were aged 16 years or older; (3) had access to a smartphone or computer (as the study included assessment of PIU); (4) reported no prior diagnosis of psychiatric disorders; and (5) provided informed consent. Participants were excluded if they (1) had incomplete responses, missing data, or extreme values (*n* = 576); (2) provided illogical or inconsistent responses (*n* = 501); and (3) had abnormal completion times (*n* = 468).

A total of 32,537 students initially participated in the survey. After data cleaning, 30,992 participants were retained for the final analysis, yielding a valid response rate of 95.25% (Figure S1).

### 2.3. Ethics Approval

This study received ethical approval from the Ethics Committee of West China Second University Hospital, Sichuan University (No. 2024048). Participation was entirely anonymous and voluntary, with informed consent obtained electronically. Before accessing the survey, participants were required to review and acknowledge an online consent form detailing the study’s purpose, confidentiality measures, and participant rights. They retained the right to withdraw at any stage without any adverse consequences.

### 2.4. Measures

#### 2.4.1. Sociodemographic Data

Sociodemographic data were collected using a self‐developed form that included the following variables: age, BMI, gender, daily exercise duration, daily screen time, residence, enrolled program, academic major, academic year, only‐child status, paternal years of education, maternal years of education, family monthly per capita income, romantic relationship status, number of romantic relationships, and sexual experience. These variables were selected based on previous research indicating their associations with psychosocial distress [25, 26].

#### 2.4.2. Psychosocial Distress

##### 2.4.2.1. Loneliness

Loneliness was assessed using the Chinese version of the UCLA Loneliness Scale (Version 3), a widely used instrument for assessing perceived social isolation [27]. The scale consists of 20 items, each rated on a 4‐point Likert scale (1 = never and 4 = always), with total scores ranging from 20 to 80. Higher scores indicate greater perceived loneliness and social isolation [27]. This scale has demonstrated good reliability and validity in the Chinese population, with a Cronbach’s *α* of 0.880 [28]. In our study, Cronbach’s *α* was 0.934.

##### 2.4.2.2. Depressive Symptoms

Depressive symptoms were evaluated using the 9‐item Patient Health Questionnaire (PHQ‐9), which assesses symptom severity over the past 2 weeks [29]. Each item was rated on a 4‐point Likert scale (0 = not at all and 3 = almost every day), with total scores between 0 and 27. Symptom severity is categorized as mild (5–9), moderate (10–14), moderately severe (15–19), and severe (20–27) based on the established cutoff points [30]. The PHQ‐9 has been validated among Chinese adolescents with a Cronbach’s *α* of 0.875 [31]. In this study, Cronbach’s *α* was 0.935.

##### 2.4.2.3. Sleep Quality

Sleep quality was measured using the Chinese version of the Pittsburgh Sleep Quality Index (PSQI), a comprehensive tool for assessing sleep quality over the past month [32]. The PSQI consists of seven aspects: subjective sleep quality (1 item), sleep latency (2 items), sleep duration (1 item), habitual sleep efficiency (3 items), sleep disturbances (9 items), sleep medication use (1 item), and daytime dysfunction (2 items). Each item is rated on a 4‐point scale from 0 (no difficulty) to 3 (severe difficulty). The total score ranged from 0 to 21, with higher scores indicating poorer sleep quality. A global score over 5 indicates impaired sleep quality, with further stratification into moderate (6–10), poor (11–15), and very poor (16–21) sleep quality [33]. Previous studies have reported a Cronbach’s *α* of 0.734 for the PSQI among Chinese university students [34]. In this study, Cronbach’s *α* was 0.713.

##### 2.4.2.4. PIU

PIU was assessed using the young internet addiction test (IAT), a widely used 20‐item self‐report scale evaluating excessive Internet use [35]. Participants rate each item using a 5‐point Likert scale (1 = rarely and 5 = always) with total scores ranging from 20 to 100. PIU severity is classified as normal (0–30), mild (31–48), moderate (50–79), and severe (80–100) [36]. The IAT has demonstrated satisfactory psychometric properties in Chinese populations, with a Cronbach’s *α* of 0.930 [37]. In this study, Cronbach’s *α* was 0.959.

#### 2.4.3. DSH and Suicidality

##### 2.4.3.1. DSH

DSH was measured using the nine‐item version of the DSH Inventory [38], adapted for adolescents by Lundh et al. [39]. The instrument evaluates eight types of DSH behaviors (e.g., cutting, burning, and scratching) and history of hospitalization. Items are rated on a 4‐point Likert scale (0 = never and 3 = more than twice), with higher scores indicating greater severity. Participants reporting any behavior more than once (i.e., scoring >1 on any item) were classified as engaging in DSH. The Chinese version of this inventory has shown good reliability, with a Cronbach’s *α* of 0.880 [40]. In this study, Cronbach’s *α* was 0.937.

##### 2.4.3.2. Suicidality

Suicidality was assessed using three items adapted from the Youth Risk Behavior Surveillance System (YRBSS) [41]. To better capture variability in the frequency and severity of suicidal thoughts and behaviors, the original binary response format was adapted to a 4‐point scale, consistent with prior research employing graded measures of suicidality [42]. The items assessed suicidal ideation, plans, and attempts over the past 12 months: (1) “During the past 12 months, how many times have you seriously considered attempting suicide?”; (2) “During the past 12 months, how many times have you made a specific plan to attempt suicide?”; and (3) “During the past 12 months, how many times have you actually attempted suicide?” Response options ranged from 0 (never) to 3 (more than twice), with higher scores indicating greater suicidality severity. The internal consistency of the measure in the current sample was high (Cronbach’s *α* = 0.911).

### 2.5. Data Collection and Quality Control

Data were collected from April to July 2024 using “Wenjuanxing,” an online survey platform widely used in China for secure and efficient data management. Participants were recruited through a convenience sampling approach, with university administrators responsible for distributing survey links and invitations to students within their institutions. A total of 32,537 responses were initially collected. To ensure data quality, a multistep validation and cleaning procedure were implemented. First, responses with missing data or extreme values were excluded (*n* = 576). Second, the built‐in logic‐check function of “Wenjuanxing” was utilized to identify and remove illogical or inconsistent responses (*n* = 501). Third, responses with abnormal completion times (i.e., outside the expected range of 10–30 min) were excluded (*n* = 468). In total, 1545 responses were removed, resulting in a final analytical sample of 30,992 participants (Figure S1).

### 2.6. Statistical Analyses

Statistical analyses were conducted using SPSS Statistics for Windows (version 25.0) and Mplus (version 8.3). All tests were two‐tailed. A nominal significance level of *α* = 0.05 was used, with adjusted thresholds being applied where appropriate.

Given the large sample size, formal normality tests (e.g., Kolmogorov–Smirnov and Shapiro–Wilk) were not emphasized due to their sensitivity to minor deviations from normality [43, 44]. Instead, distributional properties were evaluated using skewness and kurtosis. Absolute skewness > 2 and/or kurtosis > 7 were considered substantial non‐normality [43], and these indices were reported to better characterize variable distributions [45]. Continuous variables were summarized as mean ± standard deviation (SD) or median (interquartile range [IQR]) and categorical variables as frequencies and percentages. Spearman’s rank correlation was used to examine bivariate associations.

#### 2.6.1. LPA

LPA was conducted to identify distinct psychosocial distress profiles based on loneliness, depressive symptoms, sleep quality, and PIU. The optimal number of latent profiles was determined by assessing several fit indices, including the akaike information criterion (AIC), Bayesian information criterion (BIC), and sample size‐adjusted BIC (aBIC), with lower values indicating better model fit [46]. The Lo–Mendell–Rubin likelihood ratio test (LMRT) and Bootstrap likelihood ratio test (BLRT) were used to compare models with varying numbers of profiles, where a significant *p*‐value (<0.05) indicated that the *k*‐profile model was superior to the *k* − 1 profile model [47]. Entropy values, ranging from 0 to 1, were also examined, with higher values indicating better classification accuracy [47]. Profiles representing fewer than 5% of the sample were carefully scrutinized, as small profiles may lack reliability and replicability [46].

#### 2.6.2. Associations Between Different Psychosocial Distress Profiles and DSH and Suicidality

After identifying the optimal LPA solution, the Bolck–Croon–Hagenaars (BCH) method was used to examine differences in distal outcomes across profiles. A total of 14 predefined outcomes were included in the analysis, comprising one overall DSH score, nine specific DSH items, one overall suicidality score, and three individual suicidality items. This approach allows for unbiased estimation while accounting for classification uncertainty, thereby ensuring robust cross‐profile comparisons [48]. In addition, a sensitivity analysis was conducted by recoding suicidality as a binary variable (0 = no and 1 = yes; endorsement of any suicidality item) and re‐estimating the BCH models to examine the robustness of the findings.

#### 2.6.3. Sociodemographic Characteristics of Psychosocial Distress Profiles

The R3STEP function in Mplus was used to investigate associations between sociodemographic variables and profile membership via multinomial logistic regressions [49].

#### 2.6.4. Statistical Inference and Multiple Comparison Correction

For all inferential analyses, effect sizes and 95% confidence intervals (CIs) were reported alongside *p*‐values. To control for the inflation of type I error due to multiple comparisons, Bonferroni correction was applied. The 14 DSH and suicidality outcomes were treated as a single family given their conceptual relatedness and their simultaneous evaluation within the BCH framework, resulting in an adjusted significance threshold of *p* < 0.0036 (0.05/14). For sociodemographic pairwise comparisons across the three latent profiles, the adjusted threshold was set at *p* < 0.0167 (0.05/3).

## 3. Results

### 3.1. Sociodemographic Information of Participants

The final sample comprised 30,992 university students (60.57% female; *M*_age_ = 19.31 years, and SD = 1.31). Regarding demographics, 51.08% (*n* = 15,832) of participants were from rural areas, and more than half were freshmen (52.29%; *n* = 16,206). Only children comprised 28.22% (*n* = 8747) of the sample. Most participants (72.34%, *n* = 22,420) reported a family monthly per capita income of ≤5000 RMB. In addition, 22.77% (*n* = 7056) were currently in a romantic relationship, and 9.51% (*n* = 2947) reported having sexual experience. Detailed sociodemographic information is provided in Table S1.

Indicators of psychosocial distress showed mean scores of 46.03 (SD = 12.73) for loneliness, 4.34 (SD = 5.07) for depressive symptoms, 5.10 (SD = 3.55) for sleep quality, and 35.61 (SD = 15.35) for PIU. Descriptive statistics for DSH and suicidality are presented in Table 1.

**Table 1:** The characteristics of psychosocial distress, DSH, and suicidality.

Characteristics	Mean ± SD	Median (IQR)	*n* (%)	|Skewness|	|Kurtosis|
Loneliness	46.03 ± 12.73	—	—	0.62	0.20
Depressive symptoms	4.34 ± 5.07	—	—	1.54	2.99
Sleep quality	5.10 ± 3.55	—	—	0.79	0.56
PIU	35.61 ± 15.35	—	—	1.18	1.40
DSH^a,b^	1.15 ± 3.51	0.00 (0.00–0.00)	5256 (16.96)	3.98	17.93
Biting^a,b^	0.18 ± 0.57	0.00 (0.00–0.00)	1761 (5.68)	3.61	12.98
Cutting^a,b^	0.08 ± 0.37	0.00 (0.00–0.00)	3179 (10.26)	5.22	29.64
Scratching^a,b^	0.17 ± 0.56	0.00 (0.00–0.00)	3245 (10.47)	3.71	13.71
Burning^a,b^	0.17 ± 0.57	0.00 (0.00–0.00)	3245 (10.47)	3.67	13.34
Stabbing^a,b^	0.12 ± 0.47	0.00 (0.00–0.00)	2429 (7.84)	4.41	20.33
Banging^a,b^	0.11 ± 0.43	0.00 (0.00–0.00)	2181 (7.04)	4.69	23.41
Punching^a,b^	0.12 ± 0.46	0.00 (0.00–0.00)	2318 (7.48)	4.51	21.28
Other method^a,b^	0.13 ± 0.49	0.00 (0.00–0.00)	2450 (7.91)	4.35	19.48
Hospitalization for DSH^a,b^	0.07 ± 0.34	0.00 (0.00–0.00)	1571 (5.07)	5.60	34.65
Suicidality^a,b^	0.29 ± 1.12	0.00 (0.00–0.00)	2452 (7.91)	3.55	11.16
Suicidal ideation^a,b^	0.12 ± 0.46	0.00 (0.00–0.00)	2452 (7.91)	3.12	7.73
Suicidal plans^a,b^	0.09 ± 0.38	0.00 (0.00–0.00)	1796 (5.80)	3.78	12.32
Suicidal attempts^a,b^	0.08 ± 0.38	0.00 (0.00–0.00)	1752 (5.65)	3.84	12.75

*Note: n* (%) represented the proportion of participants who reported experiencing the behavior at least once during the past 12 months.

Abbreviations: DSH, deliberate self‐harm; PIU, problematic internet use.

^a^Mean and standard deviation (SD) were additionally reported to provide supporting information for key variables.

^b^Variables with substantial non‐normal distributions were additionally presented as median (IQR).

### 3.2. Subgroups Identified by LPA

#### 3.2.1. Latent Profile Model Selection

Loneliness, depressive symptoms, sleep quality, and PIU were significantly correlated in bivariate analyses (Spearman’s *ρ* = 0.26–0.66, all *p* < 0.01; Table S2), supporting the use of a person‐centered approach to capture heterogeneity in psychosocial distress profiles. Specifically, depressive symptoms showed strong correlations with sleep quality (*ρ* = 0.65) and PIU (*ρ* = 0.66), while PIU was moderately associated with loneliness (*ρ* = 0.45) and sleep quality (*ρ* = 0.48). In addition, loneliness demonstrated a moderate association with depressive symptoms (*ρ* = 0.34).

LPA models with one to five profiles were estimated, and model fit was evaluated using AIC, BIC, and aBIC (Table 2). Although the five‐profile solution demonstrated the lowest AIC, BIC, and aBIC values, one of its classes contained fewer than 5% of the sample (1.33%; *n* = 411), raising concerns regarding class stability and practical interpretability. Similarly, although the four‐profile model yielded acceptable statistical fit and high entropy, its additional class did not provide meaningful improvement in interpretability, as it primarily reflected a further subdivision within an existing severity continuum rather than a qualitatively distinct profile structure. Therefore, the three‐profile model was selected as the optimal solution based on a combination of the overall model fit, classification quality, and substantive interpretability. Specifically, this model demonstrated (1) high entropy (0.905) and significant LMR/BLRT values (*p* < 0.001), indicating classification accuracy; (2) clear clinical interpretability, with class‐specific mean levels closely aligning with established cutoff values for psychosocial distress indicators (minimal‐moderate‐severe gradation); and (3) adequate class sizes (5.20%−60.08%), ensuring stability and representativeness.

**Table 2:** Fit indices for five models using latent profile analysis (*N* = 30,992).

Model	AIC	BIC	aBIC	*p*LMR	*p*BLRT	Entropy	Smallest class (%)	Group size for each profile				
								1	2	3	4	5
1	857,967.69	858,034.42	858,008.99	—	—	—	—	30,992				
2	828,544.75	828,653.19	828,611.87	<0.001	<0.001	0.814	25.89	22,968	8024			
3	**811,840.39**	**811,990.54**	**811,933.33**	**<0.001**	**<0.001**	**0.905**	**5.20**	**18,621**	**10,759**	**1612**	—	—
4	801,912.14	802,100.00	802,030.90	<0.001	<0.001	0.897	5.04	14,831	10,147	4451	1563	—
5	796,330.49	796,564.05	796,475.06	<0.001	<0.001	0.919	1.33	14,254	10,141	4378	1808	411

*Note: p*LMR, *p*‐value for Lo–Mendell–Rubin adjusted likelihood ratio test for *k* vs. *k* −1 profiles; *p*BLRT, *p*‐value for Bootstrapped likelihood ratio test. Numbers in bold indicate optimal fit.

Abbreviations: aBIC, adjusted BIC; AIC, akaike information criterion; BIC, Bayesian information criterion.

#### 3.2.2. Characteristics of Psychosocial Distress Profiles

**Figure 1:**
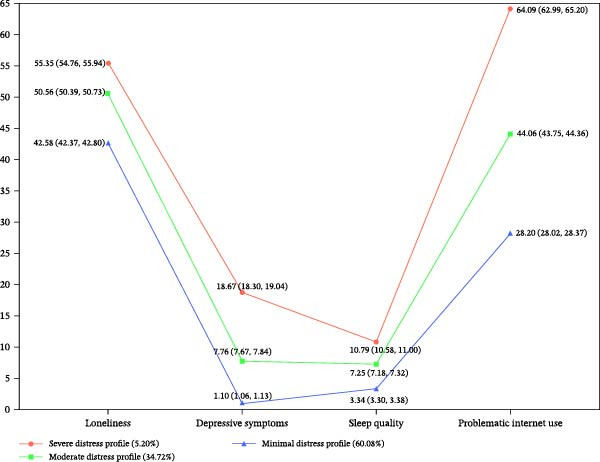
Three‐profile model of psychosocial distress. Blue indicates minimal distress profile. Green indicates moderate distress profile. Red indicates severe distress profile. Values are presented as means with 95% confidence intervals (CIs).

Three psychosocial distress profiles were identified based on mean levels of depressive symptoms, loneliness, sleep quality, and PIU (Figure 1). Profile labels were assigned with reference to established cutoff values, scale midpoints, and observed sample distributions [27, 30, 34, 50].

1. Class 1: minimal distress profile (60.08%, *n* = 18,621)
 This group exhibited the lowest levels of distress, with mean scores below the sample averages for loneliness (*M* = 42.58), depressive symptoms (*M* = 1.10), sleep quality (*M* = 3.34), and PIU (*M* = 28.20). For instance, the depressive symptom score suggests nonclinical mild symptoms, while better sleep quality and lower PIU reflect a healthy and balanced lifestyle, indicating that most individuals are at relatively low risk of psychosocial difficulties.
2. Class 2: moderate distress profile (34.72%, *n* = 10,759)
 This group exhibited moderate levels of distress, with mean scores exceeding the sample averages for loneliness (*M* = 50.56), depressive symptoms (*M* = 7.76), sleep quality (*M* = 7.25), and PIU (*M* = 44.06). These scores suggest that a significant number of individuals are experiencing moderate but potentially manageable psychosocial challenges.
3. Class 3: severe distress profile (5.20%, *n* = 1612)
 This group reported the highest levels of psychosocial distress, with mean scores exceeding both the sample averages and the midpoints of the respective scales for loneliness (*M* = 55.35), depressive symptoms (*M* = 18.67), sleep quality (*M* = 10.79), and PIU (*M* = 64.09). This group represents a relatively small but highly vulnerable subgroup, characterized by consistently elevated levels across multiple domains of psychosocial distress.

### 3.3. DSH and Suicidality Across Psychosocial Distress Profile

**Figure 2:**
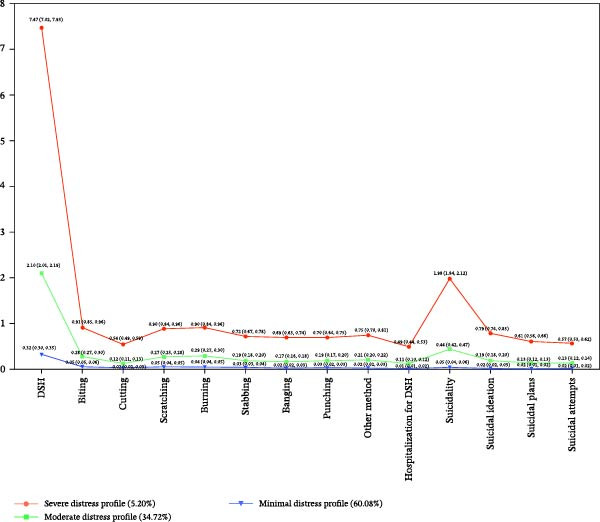
Line plots of mean differences in DSH and suicidality across psychosocial distress profiles. Blue indicates minimal distress profile. Green indicates moderate distress profile. Red indicates severe distress profile. Values are presented as BCH‐estimated means with 95% confidence intervals (CIs).

BCH‐estimated mean scores for DSH and suicidality differed significantly across psychosocial distress profiles (Table 3 and Figure 2). Participants in Class 3 exhibited the highest levels of DSH (*M* = 7.47), significantly exceeding those in Class 2 (*M* = 2.10) and Class 1 (*M* = 0.32), with all pairwise comparisons remaining significant after Bonferroni correction (*p* < 0.001). Standardized effect sizes indicated large differences between Class 3 and Class 1 (Cohen’s *d* = 2.15, 95% CI: 2.10–2.21) and moderate‐to‐large differences between Class 3 and Class 2 (*d* = 1.01, 95% CI: 0.95–1.06). Specific DSH behaviors demonstrated similar graded patterns across profiles, with Class 3 consistently showing the highest estimated mean scores. For example, biting behaviors were highest in Class 3 (*M* = 0.91), followed by Class 2 (*M* = 0.28) and Class 1 (*M* = 0.05), with large effect sizes observed between Class 3 and Class 1 (*d* = 1.85, 95% CI: 1.80–1.91).

**Table 3:** Mean differences in deliberate self‐harm and suicidality across psychosocial distress profiles (*N* = 30,992).

Outcome	Class 1	Class 2	Class 3	Pairwise comparison	Cohen’s *d* (95% CI)^a^		
					Class 1 vs. Class 2	Class 1 vs. Class 3	Class 2 vs. Class 3
DSH	0.32	2.10	7.47	Class 1＜Class 2＜Class ^∗∗∗^	0.53 (0.51, 0.55)	2.15 (2.10, 2.21)	1.01 (0.95, 1.06)
Biting	0.05	0.28	0.91	Class 1＜Class 2＜Class ^∗∗∗^	0.46 (0.43, 0.48)	1.85 (1.80, 1.91)	0.86 (0.81, 0.92)
Cutting	0.02	0.12	0.54	Class 1＜Class 2＜Class ^∗∗∗^	0.32 (0.30, 0.34)	1.53 (1.47, 1.58)	0.83 (0.78, 0.88)
Scratching	0.05	0.27	0.89	Class 1＜Class 2＜Class ^∗∗∗^	0.43 (0.41, 0.46)	1.82 (1.76, 1.87)	0.82 (0.77, 0.88)
Burning	0.04	0.29	0.9	Class 1＜Class 2＜Class ^∗∗∗^	0.47 (0.44, 0.49)	1.87 (1.81, 1.92)	0.82 (0.76, 0.87)
Stabbing	0.03	0.19	0.72	Class 1＜Class 2＜Class ^∗∗∗^	0.39 (0.36, 0.41)	1.73 (1.68, 1.79)	0.81 (0.76, 0.87)
Banging	0.02	0.17	0.69	Class 1＜Class 2＜Class ^∗∗∗^	0.40 (0.37, 0.42)	1.82 (1.77, 1.88)	0.80 (0.74, 0.85)
Punching	0.03	0.19	0.7	Class 1＜Class 2＜Class ^∗∗∗^	0.40 (0.38, 0.42)	1.75 (1.70, 1.81)	0.80 (0.75, 0.85)
Other method	0.02	0.21	0.75	Class 1＜Class 2＜Class ^∗∗∗^	0.44 (0.41, 0.46)	1.85 (1.80, 1.90)	0.79 (0.74, 0.85)
Hospitalization for DSH	0.01	0.11	0.49	Class 1＜Class 2＜Class ^∗∗∗^	0.34 (0.31, 0.36)	1.53 (1.48, 1.58)	0.78 (0.72, 0.83)
Suicidality	0.05	0.44	1.98	Class 1＜Class 2＜Class ^∗∗∗^	0.47 (0.45, 0.49)	1.94 (1.88, 1.99)	0.73 (0.68, 0.78)
Suicidal ideation	0.02	0.19	0.79	Class 1＜Class 2＜Class ^∗∗∗^	0.50 (0.47, 0.52)	2.01 (1.95, 2.06)	0.73 (0.68, 0.78)
Suicidal plans	0.02	0.13	0.61	Class 1＜Class 2＜Class ^∗∗∗^	0.40 (0.38, 0.42)	1.70 (1.65, 1.76)	0.67 (0.62, 0.73)
Suicidal attempts	0.02	0.13	0.57	Class 1＜Class 2＜Class ^∗∗∗^	0.40 (0.38, 0.43)	1.63 (1.58, 1.69)	0.62 (0.57, 0.67)

*Note:* Class 1, minimal distress profile. Class 2, moderate distress profile. Class 3, severe distress profile.

Abbreviation: DSH, deliberate self‐harm.

^a^Cohen’s *d* values with 95% confidence intervals (CIs) are reported as standardized effect sizes for pairwise comparisons. Covariates were included in the model but are not presented for clarity (refer to Table S3 for details).

^∗∗∗^All pairwise comparisons across the three profiles were statistically significant after Bonferroni correction (*p* < 0.001).

A comparable gradient was observed for suicidality outcomes. Participants in Class 3 reported the highest overall suicidality scores (*M* = 1.98), significantly exceeding those in Class 2 (*M* = 0.44) and Class 1 (*M* = 0.05) (all *p* < 0.001). The difference between Class 3 and Class 1 was associated with a large standardized effect size (*d* = 1.94, 95% CI: 1.88–1.99). Suicidal ideation, plans, and attempts showed similar graded trends across profiles.

Additionally, a sensitivity analysis using binary‐coded suicidality yielded consistent results, with a graded increase in prevalence across profiles (Class 1 < Class 2 < Class 3; all *p* < 0.001; Table S4).

### 3.4. Sociodemographic Characteristics of Psychosocial Distress Profiles

Using the minimal distress profile (Class 1) as the reference group, multinomial logistic regression analyses revealed significant associations between sociodemographic variables and distress profiles, except for age, residence, and only‐child status (Table S3).

In terms of individual and behavioral characteristics, male gender was associated with lower odds of moderate distress (OR = 0.79, 95% CI: 0.74–0.83). Longer daily exercise duration was consistently associated with lower odds of both moderate (OR = 0.82, 95% CI: 0.80–0.84) and severe distress (OR = 0.74, 95% CI: 0.70–0.79), whereas longer daily screen time was associated with increased odds of distress, with stronger effects observed in more severe profiles (Class 2: OR = 1.09, 95% CI: 1.08–1.10 and Class 3: OR = 1.17, 95% CI: 1.15–1.19).

Regarding academic and family‐related factors, associate degree enrollment was associated with higher odds of distress (Class 2: OR = 1.51, 95% CI: 1.43–1.60 and Class 3: OR = 1.53, 95% CI: 1.35–1.74). Students majoring in humanities and social sciences also exhibited elevated risk (Class 2: OR = 1.12, 95% CI: 1.04–1.20 and Class 3: OR = 1.28, 95% CI: 1.10–1.49), whereas students majoring in natural sciences were significantly associated only with moderate distress (OR = 1.10, 95% CI: 1.03–1.19). Academic year showed a consistent protective pattern, with students in earlier years demonstrating lower odds of both moderate and severe distress compared with seniors (e.g., freshman: OR = 0.77 for Class 2 and OR = 0.40 for Class 3). Regarding parental education, lower maternal education was associated with higher odds of moderate distress (≤ 6 years: OR = 1.25, 95% CI: 1.09–1.43; 7–9 years: OR = 1.22, 95% CI: 1.07–1.39; and 10–12 years: OR = 1.18, 95% CI: 1.04–1.33), whereas paternal education of 7–9 years was associated with lower odds of severe distress (OR = 0.68, 95% CI: 0.54–0.85). Lower family income, particularly ≤5000 RMB, was associated with higher odds of distress (Class 2: OR = 1.31, 95% CI: 1.18–1.46; Class 3: OR = 1.50, 95% CI: 1.22–1.84).

With respect to relationship‐related factors, being in a romantic relationship was associated with lower odds of distress (Class 2: OR = 0.89, 95% CI: 0.83–0.95 and Class 3: OR = 0.77, 95% CI: 0.67–0.89), whereas a higher number of romantic relationships (Class 2: OR = 1.07, 95% CI: 1.05–1.09 and Class 3: OR = 1.11, 95% CI: 1.07–1.16) and sexual experience (Class 2: OR = 1.21, 95% CI: 1.10–1.33 and Class 3: OR = 1.40, 95% CI: 1.18–1.67) were associated with increased odds of both moderate and severe distress.

## 4. Discussion

Based on a large nationwide sample and a person‐centered analytical approach, our study is among the first to examine multiple psychosocial challenges faced by Chinese university students. We identified three distinct psychosocial distress profiles within this population, explored their associations with the risks of DSH and suicidality, and uncovered the sociodemographic characteristics associated with high‐risk groups. These findings contribute to a more nuanced understanding of psychosocial distress in this population, providing practical implications for developing tailored mental health interventions targeting modifiable sociodemographic factors to mitigate the risks associated with DSH and suicidality.

### 4.1. Psychosocial Distress Profiles Among Chinese University Students

Our study identified three distinct psychosocial distress profiles among Chinese university students using a person‐centered analytical approach. Class 1 (60.08%) represented the lowest levels of distress and may be considered psychosocially resilient. Class 2 (34.72%) displayed moderate distress with increased emotional dysregulation. Class 3 (5.20%) was the most vulnerable group, experiencing severe distress and marked dysregulation. These findings align with previous research demonstrating heterogeneity in psychosocial functioning among university students [17, 18]. Similar to published studies, we identified a subgroup with minimal distress and another with heightened emotional dysregulation [51].

Class 2 demonstrated an intermediate level of distress, partially resembling the “Average Difficulties” or “Passive Moderate Emotion Difficulties” clusters identified in previous studies [17, 18]. In contrast, Class 3 exhibited substantially elevated levels across all psychosocial indicators, suggesting a subgroup characterized by pervasive emotional and behavioral dysregulation. This finding is consistent with prior evidence indicating that severe psychosocial distress tends to cluster across multiple domains rather than occur in isolation [52, 53]. These findings highlight the heterogeneity of psychosocial distress among university students. Importantly, by integrating multiple psychosocial indicators within a person‐centered framework, our study extends existing research and provides a more nuanced characterization of psychological vulnerability across distinct distress profiles.

### 4.2. Relationships Between Psychosocial Distress Profiles, DSH, and Suicidality

Our study identified significant associations between psychosocial distress profiles and both DSH and suicidality, with a clear graded pattern across profiles. Students in Class 3 exhibited the highest levels of DSH and suicidality, aligning with prior studies emphasizing the bidirectional relationship between severe psychosocial distress, maladaptive behaviors, and suicidal tendencies [6]. This group therefore represents the primary target for preventive and clinical interventions. In contrast, students in Class 2 showed lower levels of DSH and suicidality than those in Class 3 but remained elevated relative to Class 1. Rather than constituting a clearly defined high‐risk clinical group, Class 2 may be more appropriately interpreted as a subthreshold or transitional distress profile. Accordingly, intensive clinical intervention may warrant cautious and proportionate consideration of intervention needs. Individuals in Class 1 demonstrated the lowest levels of psychosocial distress, suggesting relatively strong emotional regulation and social support, consistent with evidence that effective emotional regulation is protective against DSH and suicidality [18, 54]. By delineating these associations, our study not only supports previous research linking increased psychosocial distress with elevated risks of suicidal ideation and behaviors among university populations [17, 19], but also contributes novel insights into the graded associations between DSH and suicidal behaviors by linking varying levels of psychosocial distress to corresponding risk levels, which may inform the development of targeted, stage‐appropriate interventions.

### 4.3. Sociodemographic Factors Associated With Variations in Psychosocial Distress

Our analysis identified several factors associated with psychosocial distress among university students. Students majoring in humanities and social sciences were more likely to report elevated psychosocial distress. This finding is consistent with previous research [55], although the underlying mechanisms remain unclear. Students who were not in romantic relationships, had a higher number of past relationships, or reported longer daily screen time were also more likely to exhibit elevated levels of psychosocial distress. These findings are consistent with prior studies suggesting that factors such as emotional support [56], relational stability [57], and digital engagement patterns [26] are associated with psychosocial distress. However, the specific pathways underlying these associations cannot be determined from the present data. In addition, students with sexual experience reported higher levels of psychosocial distress, consistent with findings from studies of South Korean adolescents [58]. Conversely, longer daily exercise duration was associated with lower levels of psychosocial distress, in line with prior research indicating a positive association between physical activity and psychosocial well‐being [25]. Senior students reported higher levels of distress compared with those in earlier academic years, which contrasts with findings from a Singapore‐based study reporting an inverse association between age and psychosocial burden [59]. Such discrepancies may reflect differences across study populations or contextual factors. Overall, these findings underscore the importance of sustained mental health support throughout the university experience to address the evolving psychosocial needs of students [60].

### 4.4. Theoretical and Clinical Implications

Our findings align with the ITS [20]. Students in Class 3 reported higher levels of loneliness and depressive symptoms, which were correlated with DSH and suicidality in the present sample. These results support existing evidence that perceived burdensomeness and thwarted belongingness may be associated with depressive symptoms, loneliness, and elevated suicidality risk. Furthermore, our study identifies distinct psychosocial distress profiles encompassing mental health and behavioral correlates among university students, offering a practical framework for conceptualizing and addressing psychosocial vulnerabilities in this population.

Clinically, our results emphasize the need for stratified mental health interventions to optimize resource allocation and prevent distress escalation. Intensive approaches such as cognitive behavioral therapy (CBT) may be particularly relevant for high‐risk students in Class 3, who presented elevated mental health concerns and maladaptive behaviors, including PIU [61]. For students in Class 2, early intervention involving stress management, resilience training, and psychoeducation may be appropriate with moderated intensity [62], while individuals in Class 1 may still benefit from wellness programs promoting physical activity, social connectedness, and mental health education [63]. Universities and policymakers should provide targeted support for younger students and design discipline‐specific interventions, especially for humanities and social science majors. Strengthening mental health education, counseling accessibility, and initiatives encouraging physical activity and healthy social relationships may help bolster psychosocial resilience. Nevertheless, the three‐profile solution may not fully capture the more nuanced heterogeneity within distress patterns. Future longitudinal studies with larger and more diverse samples are needed to validate and refine these profiles and to identify potentially distinct subprofiles that may inform more tailored and targeted intervention strategies.

### 4.5. Strengths and Limitations

A key strength of our study is its nationwide large‐scale sample and the application of LPA to identify nuanced psychosocial distress profiles among university students. To the best of our knowledge, this is the first study to explore the relationships between psychosocial stress, including mental disorders and behavioral disturbances, and suicidal risk by examining DSH across nine distinct forms and suicidality across three categories. This provides a comprehensive framework for understanding distress‐related vulnerabilities.

Several limitations should be acknowledged. First, the cross‐sectional design precludes causal inferences regarding the associations between psychosocial distress, DSH, and suicidality. In addition, it may limit the ability to fully capture the temporal dynamics and subtle heterogeneity in psychosocial distress profiles. Future longitudinal studies with repeated assessments are warranted to better examine the stability and developmental trajectories of these latent profiles over time. Second, although the nationwide scope enhances external validity, several sampling‐related limitations should be considered. The use of convenience sampling, restriction to public universities, and potential selection bias associated with online data collection may constrain full population representativeness. Public universities were selected to enhance comparability, as their institutional structures and student management practices are relatively standardized in China; however, the exclusion of private institutions may limit the generalizability of the findings. Future studies employing probability‐based sampling strategies are needed to improve generalizability and to better detect more nuanced psychosocial heterogeneity. Third, the adaptation of the YRBSS‐derived suicidality measure may introduce potential measurement limitations, as modifications from the original binary format could affect psychometric comparability. However, sensitivity analyses supported the robustness of the observed associations, suggesting acceptable stability of the findings. Future studies are warranted to further validate multidimensional assessments of suicidality in large population‐based samples. Finally, reliance on self‐reported data may introduce a reporting bias, particularly for sensitive behaviors such as self‐harm and suicidality. Future studies could enhance data validity by incorporating multisource data, including clinical assessments or objective indicators, where feasible.

## 5. Conclusion

This study identified three distinct psychosocial distress profiles among Chinese university students. Based on the selected latent profile solution, nearly 40% of participants were classified as experiencing elevated psychosocial distress. Higher levels of psychosocial distress were associated with higher levels of DSH and suicidality, highlighting the importance of promoting psychological well‐being and adaptive coping mechanisms. Students with severe psychosocial distress shared specific sociodemographic characteristics, which may help inform early identification and targeted interventions. Given the cross‐sectional design, temporal relationships cannot be established, and reverse causation cannot be ruled out. Further longitudinal and interventional studies are needed to validate these findings and support the development of effective prevention strategies.

## Funding

This study was funded by the Health Commission of Chengdu (Grant 2024007).

## Ethics Statement

This study protocol has been approved by the Ethics Committee of West China Second University Hospital, Sichuan University (Number 2024048), in compliance with the Declaration of Helsinki.

## Consent

Participation was entirely anonymous and voluntary, with informed consent obtained electronically.

## Conflicts of Interest

The authors declare no conflicts of interest.

## Supporting Information

Additional supporting information can be found online in the Supporting Information section.

## Supporting information


**Supporting Information** Figure S1: It presents the study flowchart of participant selection. Table S1: It summarizes the sociodemographic characteristics of the participants. Table S2: It shows the bivariate correlations between psychological stress variables, deliberate self‐harm (DSH), and suicidality. Table S3: It provides the results of multinomial logistic regression analyses. Table S4: It displays the results of sensitivity analyses using binary‐coded suicidality.

## Data Availability

The datasets used during the current study are available from the corresponding author upon reasonable request.
