# Supplementary material for: Psychosocial Distress Patterns and Their Associations With Deliberate Self‐Harm and Suicidality: A Latent Profile Analysis Among Chinese University Students
Source: Depress Anxiety. 2026 Jul 27;2026:4001851. doi: 10.1155/da/4001851 (PMC13403032; doi:10.1155/da/4001851)
Supplement: Supplementary file 1 — Supporting Information Figure S1: It presents the study flowchart of participant selection. Table S1: It summarizes the sociodemographic characteristics of the participants. Table S2: It shows the bivariate correlations between psychological stress variables, deliberate self‐harm (DSH), and suicidality. Table S3: It provides the results of multinomial logistic regression analyses. Table S4: It displays the results of sensitivity analyses using binary‐coded suicidality. [file DA-2026-4001851-s001.docx]

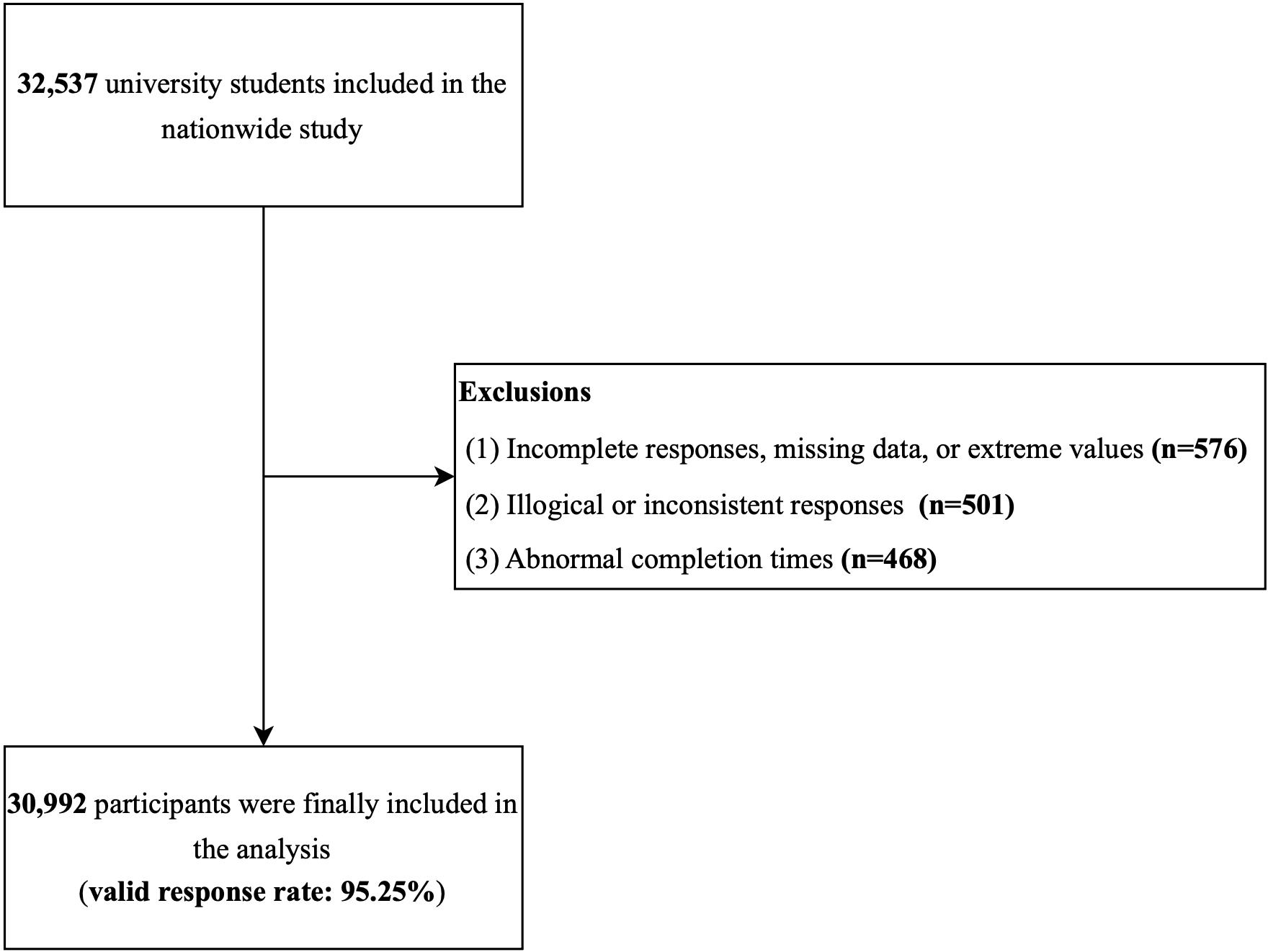


***Figure S1.* Flowchart of participant recruitment and selection (final analytic sample: *N* = 30,992).**

***Table S1.* Sociodemographic information of participants (*N* = 30,992).**

| **Characteristics** | **Mean ± SD or n (%)** |
| --- | --- |
| Age | 19.31±1.31 |
| Weight | 59.36±12.57 |
| BMI | 21.42±4.20 |
| Gender |  |
| *Male* | 12220 (39.43%) |
| *Female* | 18772 (60.57%) |
| Daily exercise duration | 1.19±1.05 |
| Daily screen time | 6.27±3.24 |
| Residence |  |
| *Urban/Town* | 15160 (48.92%) |
| *Rural* | 15832 (51.08%) |
| Enrolled program |  |
| *Associate degree* | 16537 (53.36%) |
| *Bachelor's degree* | 14455 (46.64%) |
| Academic major |  |
| *Humanities and social sciences* | 11865 (38.28%) |
| *Natural sciences* | 12101 (39.05%) |
| *Medical sciences* | 7026 (22.67%) |
| Academic year |  |
| *Freshman* | 16206 (52.29%) |
| *Sophomore* | 8931 (28.82%) |
| *Junior* | 4261 (13.75%) |
| *Senior* | 1594 (5.14%) |
| Only-child status |  |
| *Yes* | 8747 (28.22%) |
| *No* | 22245 (71.78%) |
| Paternal years of education |  |
| ≤6 | 8085 (26.09%) |
| 7-9 | 12697 (40.96%) |
| 10-12 | 7229 (23.33%) |
| ＞12 | 2981 (9.62%) |
| Maternal years of education |  |
| ≤6 | 10900 (35.17%) |
| 7-9 | 11685 (37.71%) |
| 10-12 | 6110 (19.71%) |
| ＞12 | 2297 (7.41%) |
| Family monthly per capita income |  |
| ≤5000 | 22420 (72.34%) |
| 5001-10000 | 5941 (19.17%) |
| ＞10000 | 2631 (8.49%) |
| Romantic relationship status |  |
| *In a relationship* | 7056 (22.77%) |
| *Not in a relationship* | 23936 (77.23%) |
| Number of romantic relationships | 1.12±1.40 |
| Sexual experience |  |
| *Yes* | 2947 (9.51%) |
| *No* | 28045 (90.49%) |

***Table S2.* Bivariate correlations between psychological stress variables, DSH, and suicidality (*N* = 30,992).**

|  | **Loneliness** | **Depressive symptoms** | **Sleep quality** | **PIU** | **DSH** | **DSH1** | **DSH2** | **DSH3** | **DSH4** | **DSH5** | **DSH6** | **DSH7** | **DSH8** | **DSH9** | **S** | **S1** | **S2** | **S3** |
| --- | --- | --- | --- | --- | --- | --- | --- | --- | --- | --- | --- | --- | --- | --- | --- | --- | --- | --- |
| **Loneliness** | 1 |  |  |  |  |  |  |  |  |  |  |  |  |  |  |  |  |  |
| **Depressive symptoms** | 0.34** | 1 |  |  |  |  |  |  |  |  |  |  |  |  |  |  |  |  |
| **Sleep quality** | 0.26** | 0.65** | 1 |  |  |  |  |  |  |  |  |  |  |  |  |  |  |  |
| **PIU** | 0.45** | 0.66** | 0.48** | 1 |  |  |  |  |  |  |  |  |  |  |  |  |  |  |
| **DSH** | 0.14** | 0.38** | 0.28** | 0.32** | 1 |  |  |  |  |  |  |  |  |  |  |  |  |  |
| **DSH1** | 0.10** | 0.31** | 0.22** | 0.26** | 0.81** | 1 |  |  |  |  |  |  |  |  |  |  |  |  |
| **DSH2** | 0.01* | 0.23** | 0.13** | 0.21** | 0.60** | 0.64** | 1 |  |  |  |  |  |  |  |  |  |  |  |
| **DSH3** | 0.09** | 0.31** | 0.22** | 0.26** | 0.79** | 0.85** | 0.67** | 1 |  |  |  |  |  |  |  |  |  |  |
| **DSH4** | 0.10** | 0.32** | 0.22** | 0.28** | 0.79** | 0.66** | 0.65** | 0.68** | 1 |  |  |  |  |  |  |  |  |  |
| **DSH5** | 0.06** | 0.28** | 0.18** | 0.24** | 0.70** | 0.71** | 0.71** | 0.73** | 0.65** | 1 |  |  |  |  |  |  |  |  |
| **DSH6** | 0.05** | 0.28** | 0.18** | 0.24** | 0.66** | 0.63** | 0.73** | 0.65** | 0.65** | 0.71** | 1 |  |  |  |  |  |  |  |
| **DSH7** | 0.06** | 0.28** | 0.18** | 0.24** | 0.68** | 0.62** | 0.70** | 0.64** | 0.65** | 0.70** | 0.78** | 1 |  |  |  |  |  |  |
| **DSH8** | 0.08** | 0.30** | 0.20** | 0.26** | 0.70** | 0.64** | 0.67** | 0.65** | 0.67** | 0.71** | 0.72** | 0.71** | 1 |  |  |  |  |  |
| **DSH9** | 0.01* | 0.24** | 0.14** | 0.20** | 0.56** | 0.60** | 0.79** | 0.62** | 0.60** | 0.70** | 0.74** | 0.72** | 0.70** | 1 |  |  |  |  |
| **S** | 0.06** | 0.31** | 0.20** | 0.26** | 0.61** | 0.60** | 0.66** | 0.62** | 0.60** | 0.66** | 0.67** | 0.67** | 0.68** | 0.72** | 1 |  |  |  |
| **S1** | 0.07** | 0.31** | 0.20** | 0.25** | 0.60** | 0.59** | 0.65** | 0.61** | 0.59** | 0.64** | 0.65** | 0.65** | 0.66** | 0.70** | 0.97** | 1 |  |  |
| **S2** | 0.02** | 0.26** | 0.16** | 0.22** | 0.57** | 0.60** | 0.75** | 0.62** | 0.60** | 0.69** | 0.71** | 0.69** | 0.69** | 0.82** | 0.84** | 0.79** | 1 |  |
| **S3** | 0.02** | 0.26** | 0.15** | 0.22** | 0.56** | 0.60** | 0.75** | 0.62** | 0.60** | 0.69** | 0.71** | 0.69** | 0.70** | 0.82** | 0.83** | 0.78** | 0.86** | 1 |

Notes: Spearman’s rank correlation coefficients were calculated due to the non-normal distributions of several variables. **p*＜0.05; ***p*＜0.01. PIU = problematic internet use; DSH = deliberate self-harm; DSH1 = biting; DSH2 = cutting; DSH3 = scratching; DSH4 = burning; DSH5 = stabbing; DSH6 = banging; DSH7 = punching; DSH8 = other methods; DSH9 = hospitalization for DSH; S = suicidality; S1 = suicidal ideation; S2 = suicidal plans; S3 = suicidal attempts.

***Table S3.* The results of multinomial logistic regression (*N* = 30,992).**

| **Covariates** | **Class 1^#^**  (n=18,621) | | | **Class 2**  (n=10,759) | | | **Class 3**  (n=1,612) | | |
| --- | --- | --- | --- | --- | --- | --- | --- | --- | --- |
|  | OR | 95% CI | *p* | OR | 95% CI | *p* | OR | 95% CI | *p* |
| Age | - | - | - | 0.99 | (0.96, 1.02) | 0.43 | 0.95 | (0.89, 1.00) | 0.05 |
| BMI | - | - | - | 1.00 | (1.00, 1.01) | 0.29 | 1.00 | (0.99, 1.01) | 0.87 |
| Gender | - | - | - |  |  |  |  |  |  |
| *Male* |  |  |  | 0.79 | (0.74, 0.83) | ＜0.001** | 1.01 | (0.90, 1.14) | 0.84 |
| *Female* ^#^ |  |  |  | - | - | - | - | - | - |
| Daily exercise duration | - | - | - | 0.82 | (0.80, 0.84) | ＜0.001** | 0.74 | (0.70, 0.79) | ＜0.001** |
| Daily screen time | - | - | - | 1.09 | (1.08, 1.10) | ＜0.001** | 1.17 | (1.15, 1.19) | ＜0.001** |
| Residence | - | - | - |  |  |  |  |  |  |
| *Urban/Town* |  |  |  | 1.00 | (0.94, 1.06) | 0.92 | 0.98 | (0.87, 1.11) | 0.75 |
| *Rural* ^#^ |  |  |  | - | - | - | - | - | - |
| Enrolled program | - | - | - |  |  |  |  |  |  |
| *Associate degree* |  |  |  | 1.51 | (1.43, 1.60) | ＜0.001** | 1.53 | (1.35, 1.74) | ＜0.001** |
| *Bachelor's degree* ^#^ |  |  |  | - | - | - | - | - | - |
| Major | - | - | - |  |  |  |  |  |  |
| *Humanities and social sciences* |  |  |  | 1.12 | (1.04, 1.20) | ＜0.001** | 1.28 | (1.10, 1.49) | ＜0.001** |
| *Natural sciences* |  |  |  | 1.10 | (1.03, 1.19) | 0.01* | 1.09 | (0.93, 1.29) | 0.28 |
| *Medical sciences* ^#^ |  |  |  | - | - | - | - | - | - |
| Academic year | - | - | - |  |  |  |  |  |  |
| *Freshman* |  |  |  | 0.77 | (0.67, 0.88) | ＜0.001** | 0.40 | (0.30, 0.52) | ＜0.001** |
| *Sophomore* |  |  |  | 0.81 | (0.71, 0.92) | ＜0.001** | 0.57 | (0.45, 0.72) | ＜0.001** |
| *Junior* |  |  |  | 0.88 | (0.78, 1.00) | 0.06 | 0.74 | (0.59, 0.92) | 0.01* |
| *Senior* ^#^ |  |  |  | - | - | - | - | - | - |
| Only-child status | - | - | - |  |  |  |  |  |  |
| *Yes* |  |  |  | 1.02 | (0.96, 1.08) | 0.51 | 0.99 | (0.88, 1.12) | 0.91 |
| *No* ^#^ |  |  |  | - | - | - | - | - | - |
| Paternal years of education | - | - | - |  |  |  |  |  |  |
| ≤6 |  |  |  | 1.05 | (0.93, 1.19) | 0.44 | 0.85 | (0.67, 1.09) | 0.20 |
| 7-9 |  |  |  | 1.04 | (0.93, 1.16) | 0.51 | 0.68 | (0.54, 0.85) | ＜0.001** |
| 10-12 |  |  |  | 1.02 | (0.92, 1.14) | 0.70 | 0.82 | (0.66, 1.01) | 0.06 |
| ＞12 ^#^ |  |  |  | - | - | - | - | - | - |
| Maternal years of education | - | - | - |  |  |  |  |  |  |
| ≤6 |  |  |  | 1.25 | (1.09, 1.43) | ＜0.001** | 1.15 | (0.88, 1.49) | 0.32 |
| 7-9 |  |  |  | 1.22 | (1.07, 1.39) | ＜0.001** | 1.04 | (0.80, 1.33) | 0.79 |
| 10-12 |  |  |  | 1.18 | (1.04, 1.33) | 0.01* | 1.11 | (0.88, 1.41) | 0.38 |
| ＞12 ^#^ |  |  |  | - | - | - | - | - | - |
| Family monthly per capita income | - | - | - |  |  |  |  |  |  |
| ≤5000 |  |  |  | 1.31 | (1.18, 1.46) | ＜0.001** | 1.50 | (1.22, 1.84) | ＜0.001** |
| 5001-10000 |  |  |  | 1.24 | (1.12, 1.37) | ＜0.001** | 1.09 | (0.89, 1.33) | 0.43 |
| ＞10000 ^#^ |  |  |  | - | - | - | - | - | - |
| Romantic relationship status | - | - | - |  |  |  |  |  |  |
| *In a relationship* |  |  |  | 0.89 | (0.83, 0.95) | ＜0.001** | 0.77 | (0.67, 0.89) | ＜0.001** |
| *Not in a relationship* ^#^ |  |  |  | - | - | - | - | - | - |
| Number of romantic relationships | - | - | - | 1.07 | (1.05, 1.09) | ＜0.001** | 1.11 | (1.07, 1.16) | ＜0.001** |
| Sexual experience | - | - | - |  |  |  |  |  |  |
| *Yes* |  |  |  | 1.21 | (1.10, 1.33) | ＜0.001** | 1.40 | (1.18, 1.67) | ＜0.001** |
| *No* ^#^ |  |  |  | - | - | - | - | - | - |

OR = Odds ratio; ^#^ Reference group; **p*＜0.05; ***p*＜0.01. Class 1: *minimal distress profile*. Class 2: *moderate distress profile*. Class 3: *severe distress profile*.

***Table S4.*** Prevalence of suicidality and its components across psychosocial distress profiles (binary classification, sensitivity analysis) (N = 30,992).

| **Outcome** | **Class 1** | **Class 2** | **Class 3** | **Pairwise comparison** |
| --- | --- | --- | --- | --- |
| **Suicidality** | 0.017 | 0.148 | 0.428 | Class 1＜Class 2＜Class 3*** |
| Suicidal ideation | 0.015 | 0.140 | 0.411 | Class 1＜Class 2＜Class 3*** |
| Suicidal plans | 0.012 | 0.098 | 0.319 | Class 1＜Class 2＜Class 3*** |
| Suicidal attempts | 0.011 | 0.098 | 0.300 | Class 1＜Class 2＜Class 3*** |

Notes: Values represent BCH-adjusted estimated probabilities (prevalence) of endorsing each suicidality outcome (0 = no, 1 = yes) within each latent profile, accounting for classification uncertainty. Differences across profiles were examined using the BCH method in Mplus. Class 1: *minimal distress profile*. Class 2: *moderate distress profile*. Class 3: *severe distress profile*. ****p*＜0.001.
